# Mental and somatic health complaints associated with school bullying between 10^th ^and 12^th ^grade students; results from cross sectional studies in Oslo, Norway

**DOI:** 10.1186/1745-0179-5-6

**Published:** 2009-03-23

**Authors:** Lars Lien, Kristian Green, Audun Welander-Vatn, Espen Bjertness

**Affiliations:** 1 Institute of Psychiatry, University of Oslo, Box 1130, 0318 Oslo, Norway; 2 Innlandet Hospital Trust HF, DPS Gjøvik, Kyrre Grepps 22, 2819 Gjøvik, Norway; 3 Institute of General Practice and Community Medicine, University of Oslo, Box 1130, 0318 Oslo, Norway; 4 Tibet University Medical College, 24 Niangre Rd, Lhasa, Tibet Autonomous Region, PR China

## Abstract

**Background:**

Bullying is a widespread and serious problem that might influence both mental and psychical well being as well as school performance and social life. The aim of this study was to describe the prevalence of bullying, mental health problems and psychical complaints among 10^th ^and 12^th ^grade students and to analyze the association between bullying, mental health problems and muscle and skeletal complaints.

**Methods:**

Two cross sectional studies of adolescents living in Oslo, Norway the first conducted in 2001 among 10^th ^grade students (15/16 years old) and the second in 2004 among 12^th ^grade students (18/19 years old). Both surveys were based on self report, were mostly school based and had almost identical questionnaires. There were around 3700 participants in both surveys, but the participation rate was lower in the latter survey (88 versus 80%). The Hopkins Symptoms Check List (HSCL-10) and the Strength and Difficulties Questionnaire (SDQ) were used to measure mental health problems.

**Results:**

Bullying is decreasing both among boys and girls while the prevalence of internalized mental health problems are increasing from 10^th ^to 12^th ^grade. For muscle and skeletal pain there is a diverging trend between boys and girls, with an increase among girls and a decrease among boys. The highest Odds Ratios, as a measure for the association between bullying, mental health problems and pain, were found for internalized mental health problems at both 10^th ^and 12^th ^grade both for boys and girls.

**Conclusion:**

Both internalized and externalized mental health problems together with pain seem to be associated with bullying irrespective of school type and gender.

## Background

Bullying is a serious and widespread problem among children and adolescents, and a traumatic life event that can have serious potential consequences for mental and physical health [1-4]. Several studies indicate an association with both mental and physical health with the same strong associations as for other assaults like sexual assault experienced by some children and adolescents [5].

The prevalence of bullying varies with study place and design and comparisons are difficult due to lack of uniform criteria and differences in study design and sample. One exception is the series of cross sectional studies of 11 to 15 years old pupils with participation of 28 European countries using standardized procedures and criteria [6]. There is great variation between countries with the lowest prevalence found in Sweden where around 6% had experienced bullying and the highest was found in Lithuania where an average of around 40% reported being bullied during the last year [6].

Some studies indicate that more boys than girls are affected by bullying [1,6-10], but the differences are in general small [1,6-10]. In Norway 11% of the girls and 15% of the boys reported bullying in the abovementioned European study [6]. There are significant differences in type of bullying among boys and girls. While boys are mostly affected by direct bullying such as intimidations, abusive acts and direct violent assaults indirect or relational bullying such as social isolation, ignoring and spreading of rumors is more common among girls [11,12].

In the past bullying was looked upon as a transient and harmless act without serious consequences for those involved [11]. Today, however, bullying is found to be associated with psychosocial and somatic illness, especially when the bullying is frequent and long standing [3-7]. The main focus of research has been on the association between bullying and mental health problems where level of anxiety, depression, loneliness, self confidence, suicidal behavior and behavioral problems have been found to be associated both with victimization of bullying and being the one bullying [1,10,13-16]. Recently a dose-response association between bullying and sub-clinical psychotic symptoms was found among Dutch 14 years old adolescents [5].

Several studies have also found associations between bullying and somatic symptoms like headache, sleep disturbances, stomach pain, enuresis, dizziness, common cold, and musculoskeletal tenderness and pain [3,17,18]. Longitudinal studies support the hypothesis that bullying causes ill health and not the other way around [2,7,8,19-21]. Studies also indicate that bullying during adolescence increases the risk for later mental health problems [22,23].

Bullying is more prevalent in the young adolescent groups and the prevalence drops with increasing age for both boys and girls [6,8,19,20,24]. Bullying, however, is not a phenomenon that only takes place in early adolescence, but can be found among children and in late adolescence as well [21]. There even seem to be some individuals that are vulnerable to bullying throughout childhood, adolescence and further into adulthood [24,25].

The aims of this study are therefore to:

1. Describe the prevalence of bullying, mental health problems and muscle and skeletal complaints among 15/16 and 18/19 year old adolescents.

2. Analyze the association between bullying, mental health problems and muscle and skeletal complaints among adolescents aged 15/16 and 18/19 years.

3. Assess the possible dose-response relationship between bullying and both mental health problems and muscle and skeletal complaints among 15/16 year old adolescents.

## Materials and methods

### Data source and subject selection

Two surveys among adolescents in Oslo, Norway are the data sources for this study; the Youth part of the Oslo Health Study 2000–2001 (HUBRO) and Youth 2004. Both surveys were joint collaboration projects where the Norwegian Institute of Public Health and the University of Oslo were partners in both projects with the Municipality of Oslo in the HUBRO study and with the Regional Centre for Child and Adolescent Psychiatry in Youth 2004.

In the HUBRO study all pupils in the tenth grade of all schools in Oslo during the years 2000 and 2001 were included, and they completed an eight pages questionnaire. The tenth grade is compulsory in Norway and, the survey therefore included two cohorts of all 15/16-year olds. The "Youth 2004" study, which is building on the 2001-cohort of the youth part of HUBRO, was conducted in 2004 at all Upper Secondary (12^th ^grade) schools in Oslo among 18/19 year olds. In this study a four pages questionnaire was completed with most of the questions being similar in the two studies. Both studies were conducted during school hours. In addition, students participating in 2001 that were not enrolled in the terminal year at Upper Secondary school, received survey material by post in the "Youth 2004" study. More information about the youth part of HUBRO can be found at: http://www.fhi.no/dav/831c96A203.doc and for the "Youth 2004" at: http://www.fhi.no/dav/A34847D246.pdf

There were originally 7343 participants in the youth part of HUBRO. For the purpose of this study we included only the 2001 cohort to have equal number of participants the two groups. The 2001 cohort had 3811 participants, but for 21 there were unknown information about gender due to data-error. These 21 were then excluded, resulting in 3790 participants with a participation rate of 89% among boys and 92% among girls. The corresponding figures for the "Youth 2004" part of the study were 3790 with a participation rate of 73% among boys and 86% among girls. Of the participants in the latter study 15% came from the mail part of the survey, giving a participation rate in this group of 34%. Compared to the HUBRO part of the study, the Youth 2004 has fewer boys and fewer immigrants from non-western countries.

### The questionnaires

The questionnaires used in the two studies differed slightly. Most of the questions in the Youth 2004 study (used among the 18/19 years old) are the same as that used in the HUBRO study (15/16 years), except that the wording differs somewhat for some questions, like for that on bullying (see below). In Youth 2004 there was an added focus on mental health, physical activity and reading and writing disabilities. The projects dealing with these topics were the main reason for the implementation of Youth 2004. In addition to the topics mentioned, the questionnaire covers the following subjects: self-reported health, stress, coping, social support, education and educational plans, alcohol and smoking habits, antisocial behaviour, nutrition and weight loss, sexual behaviour and use of contraceptives, use of medicines, the utilization of health services and skin problems.

For both surveys it was emphasized that the questions should be validated and preferably used in previous youth surveys. For the HUBRO study two 4-page questionnaires (named U and U/T) were designed. All questions on questionnaire U/T were suggested and paid by associated researchers. They had projects presented for and accepted by the board of HUBRO. Questionnaires included questions concerning health, physical activity, smoking, intoxicants, use of medicines, sexual behaviour and contraception, food and drink, education and plans for the future, adolescence and sense of belonging. There was also a focus on strong and weak sides, concerns, the situation at school, culture and contact, relationship to family and friends, mourning and war experiences. For further information on the questions and to see the whole questionnaire see: http://www.fhi.no/eway/default.aspx?pid=238&trg=MainLeft_5895&MainArea_5811=5895:0:15,4225:1:0:0:::0:0&MainLeft_5895=5825:28244::1:5830:3:::0:0

### Outcome variables

#### 1. Internalizing mental health problems

Internalizing mental health problems were measured by the ten-item version of Hopkins Symptoms Check List (HSCL-10). The 10 items included in this short version are; feeling panicky, anxious, dizzy, tense, sleepless, sad, worthless, hopeless, fault within self and finding everything a burden, all during the past week. The internal reliability was high (Cronbach α: .86 at age 15/16 in the present data material). Each item is rated on a scale of 1 (not at all) to 4 (extremely). An average score for all 10 items of equal or above 1.85 has shown to be a valid predictor for mental distress among subjects aged 16–24 year of age, corresponding to the 1.75 cut-off of HSCL-25 [26].

#### 2. Externalizing mental health problems

SDQ is a questionnaire for assessing mental health in children and adolescents with five subscales; emotional problems, conduct problems, hyperactivity and peer problems adding up to a total difficulties score. In addition there is a positive prosocial score. The rating scale for SDQ is from 1 to 3 with the options of "not correct", "partly correct" and "completely correct". For the purpose of this study we used only two of the subscales. Externalizing mental health problems were measured by ten items about hyperactivity and conduct problems from the Strength and Difficulties Questionnaire (SDQ) [27,28]. As a cut-off point we used the 90^th ^percentile of the youth part of the Oslo Health Study, which has been previously applied in other studies [29].

#### 3. Muscle and skeletal pain

Muscle and skeletal pain were measured by the following questions: **"**Have you in the last 12 months experienced pain several times in: head, neck/shoulder, arms/legs/knees, stomach, back?"; with responses being "yes" or "no". On the basis of these answers we grouped the adolescents in three groups; 0 pain sites, 1–2 pain sites and 3 to 5 pain sites, treating all pain sites with equal weight [30].

### Exposure variables

To assess exposure to bullying, the 15/16 year olds responded to the item: "Have you, in the course of the last 12 months experienced bullying at school/on the way to school?", with the response categories were "never", "sometimes", "about once a week", and "several times a week". The 18/19 year olds were asked: "Have you since 10^th ^grade experienced bullying, with the response options "no", "yes, and "yes, during the last 12 months".

### Background variables

The background variables are selected on the basis of their known association both to mental and somatic health problems and bullying. Among the immigrant adolescents, a majority was born in Norway and are second generation immigrants. Minority status was therefore determined on the basis of their parents' country of birth. In this study, we applied the Statistics Norway's definition of immigrants (or ethnic minorities), namely having both parents born in a country other than Norway.

Data on family structure was obtained from the participants' response to the item: "Who do you live together with at present?" We categorized their responses into "both parents" (corresponding to having marked "mother and father"), "one parent" (including the responses "mother only", "father only", "about the same time with mother and father", and "mother or father and new partner or husband/wife"), "foster parents", and "other".

Self-perceived socioeconomic status was obtained from the participants' response to the item "I think that our family, seen in relation to other families in Norway, has: poor, moderate, good, or very good economy".

To obtain information on close friends, the participants responded to the statement: "I have one or more close friends" with either "not true", "partly true" or "completely true".

The following question was used to register exposure to violence: "Have you been exposed to violence during the last 12 months?" The response categories were "never", "yes (by youths only"), "yes (by adults only)", and "yes (by both youths and adults")

### Statistical methods

The Statistical Package for the Social Sciences (SPSS for Windows, version12, SPSS Inc., Chicago, IL, USA) was used for the data analyses. The same strategy of analysis was used in both cross sectional studies. Cross tables were analyzed with Pearson's Chi square test to compare genders on the prevalence of bullying, mental health problems and muscle and skeletal complaints. To analyze the association between exposure to bullying and the outcome variables, a logistic regression model, with exposure to bullying as the independent variable and the outcome variables as dependent variables, was used to estimate both crude odds ratios and adjusted odds ratios in multivariate analyses. In the multivariate analyses the variables for adjustment were exposure to violence, number of close friends, ethnicity, family structure, and socio-economic status (SES). The analyses were stratified by gender. The level of significance was set to p ≤ 0.05.

## Results

### Descriptive statistics

The gender distribution was 50.7% boys and 49.3% girls in the age group of 15/16 years, compared to 44.1% boys and 55.9% girls in the age group of 18/19 years (Table 1). Among the boys, 24.1% of the 15/16 year olds and 20% of the 18/19 year olds had both their parents born outside Norway, while the comparable figures for girls were 24.6% and 23.1%, respectively. The majority of the participants lived with both of their parents, while more than one out of four lived with one parent only. A higher proportion of the 18/19 years old adolescents perceived that there families had poor family economic status compared to the 15/16 to years age group.

**Table 1:** Characteristics of the samples

	Boys				Girls			
	2001 (N = 1923)		2004 (N = 1670)		2001 (N = 1867)		2004 (N = 2120)	
	N	%	N	%	N	%	N	%
Parents' country of birth								
At least one from Norway	1438	75,9	1000	80,0	1395	75,4	1203	76,9
Both from other country	456	24,1	250	20,0	456	24,6	362	23,1
Living with								
Both parents	1329	70,0	983	62,4	1231	66,4	1163	57,4
One parent	550	29,0	425	27,0	597	32,2	539	26,6
Foster parents	5	0,3	4	0,3	11	0,6	11	0,5
Other	15	0,8	164	10,4	15	0,8	314	15,5
Socioeconomic status								
Poor	53	2,8	73	4,6	59	3,2	102	5,1
Moderate	486	25,7	434	27,6	560	30,6	643	31,9
Good	1042	55,2	824	52,5	1005	54,9	1036	51,3
Very good	307	16,3	239	15,2	205	11,2	237	11,7
Exposed to bullying in 2001								
Never	1590	83,8			1592	85,6		
Sometimes	235	12,4			217	11,7		
Weekly	33	1,7			16	0,9		
Several times a week	40	2,1			34	1,8		
Exposed to bullying in 2004								
No			1529	94,2			1933	93,7
Yes, not last 12 months			76	4,7			85	4,3
Yes, incl. last 12 months			18	1,1			41	2,0

In the age group 15/16 years, 12.4% of the boys and 11.7% of the girls had been bullied sometimes, while 3.8% of the boys and 2.7% of the girls had been bullied weekly or several times a week (Table 1). Among the 18/19 year olds, 4.7% of the boys and 4.3% of the girls had been bullied after 10^th ^grade. In the same age group, the prevalence of exposure to bullying during the last 12 months was 1.1% among boys and 2.0% among girls. Pearson's Chi square tests revealed no statistically significant differences between the genders in the prevalence of bullying neither among 15/16 year olds (p = 0,08) nor 18/19 year olds (p = 0,10).

Internalized mental health problems above our cut-off were reported by 9.7% of the boys and 26.7% of the girls aged 15/16 (Additional file 1, Table S1). In the age group of 18/19, the comparable figures were 14.0% and 34.5%. There was a tendency that more boys than girls scored above cut-off on externalized mental health problems in the youngest age group; 14.1% compared to 10.4%, while in the older age group both boys and girls had a prevalence of approximately 9%.

Among the pain complaints, headache was the most frequent one, with a prevalence of 68.0% among girls aged 18/19. Boys in the same age group had a prevalence of 39.0%, which was lower than the 45.8% in the younger age group. Girls had higher prevalence of abdominal pain and neck/shoulder pain, with the most pronounced sex differences found for abdominal pain; 20.5% vs. 49.7% among 15/16 year olds and 14.4% vs. 47.4% in the other age group.

### Multivariate logistic regression analyses of the association between exposure to bullying and health complaints among 15/16 year olds

Multivariate logistic regression analyses among 15/16 year olds showed significant associations between exposure to bullying and reporting internalized mental health problems (Additional file 1, Table S2). Among boys, the crude odds ratio (OR) 3.4 for "exposed to bullying sometimes" compared with the reference category "never bullied", while the OR for "exposed to bullying several times a week" was 13.3. When adjusting for exposure to violence, having close friends, ethnicity, family structure, and socio-economic status (SES), the odds ratios decreased, but were still statistically significant. Among girls, reporting bullying weekly gave the highest crude OR of 4.7, while exposure to bullying sometimes or several times a week had crude OR of 2.0 and 3.1, respectively.

Reporting externalized mental health problems was associated with exposure to bullying among boys, with crude OR of 4.0 for weekly exposure to bullying; however, only exposure several times a week was statistically significant when adjusting for ethnicity, family structure, and SES. Among girls, exposure to bullying several times a week was associated with externalized problems, but this association was not statistically significant in the multivariate model.

We found statistically significant crude associations between exposure to bullying and all pain categories, though not for all categories of exposure to bullying. Headache was associated with exposure to bullying sometimes and several times a week for both sexes, while only "bullying sometimes" was significant in the multivariate model. The crude OR for neck/shoulder pain in boys were 4.6 for weekly bullying and 3.1 for bullying several times a week; these figures were marginally lower in the multivariate model. In girls, neck/shoulder pain as well as pain in arm, leg or knee were associated with "bullied sometimes", but not with bullying weekly or several times a week.

In boys, pain in arm, leg or knee was associated with all levels of exposure to bullying, with crude OR ranging from 1.6 to 3.5, while "bullying several times a week" was not statistically significant in the multivariate model. Abdominal pain and back pain showed a dose-response pattern in boys, with crude OR for back pain increasing from 1.7 for "bullied sometimes" via 2.2 for "bullied weekly" to 6.1 for "bullied several times a week". When adjusting for background factors, however, the associations between "sometimes bullied" and abdominal pain, and between "weekly bullied" and back pain, were no longer statistically significant. In girls, reporting bullying "sometimes" was associated with abdominal pain and back pain, and the estimates from the multivariate model differed only marginally from the crude estimates.

### Multivariate logistic regression analyses of the association between exposure to bullying and health complaints among 18/19 year olds

In the multivariate logistic regression analyses among 18/19 year olds, we found significant associations between exposure to bullying and reporting internalized mental health problems, with crude OR of 4.4; 2.7–7.3 for "bullied, but not the last 12 months" and OR 7.1; 2.8–18.1 for "bullied during the last 12 months" for boys (Additional file 1, Table S3). When adjusting for ethnicity, family structure, and socio-economic status (SES), only "bullied, but not last 12 months" remained statistically significant. For girls, the comparable crude OR were 4.8 for "bullied but not last 12 months" and 4.6 for "bullied during the last 12 months", with only minor changes in the estimated OR in the multivariate model. Externalized mental health problems were associated with "bullied but not last 12 months" and with "bullied during the last 12 months".

Among girls externalized mental health problems were associated with "bullied, but not last 12 months" only. We found associations among boys between exposure to bullying and all pain sites except for abdominal pain, but these associations were not statistically significant in the multivariate models. The lowest crude OR was obtained for the association between headache and "bullied, but not last 12 months", which was 1.8 and the highest crude OR was observed for the association between pain in arm/leg/knee and "bullied during the last 12 months", which was 9.5. Among girls, exposure to bullying was associated with all pain sites except headache, with crude OR varying from 1.9 to 2.5 for the different pain sites. In the multivariate model, only two finding remained statistically significant; namely the associations between pain in arm/leg/knee and "bullied during the last 12 months" and between abdominal pain and "bullied, but not last 12 months".

## Discussion

In this study of bullying and the association with mental health problems and muscle and skeletal pain among both 15/16 years and 18/19 years old students the following findings were most prominent:

1. Bullying seems to decrease both among boys and girls from 15/16 years of age to 17/18 (those being bullied since 15/16 years of age, but not the last year) and with a further decline among the 18/19 years old adolescents. This is in line with other studies showing that the prevalence of bullying is reduced with increasing age [3,6,8,19,24,31]. This might, however, also be due to possible selection bias among the 18/19 years old in our study.

2. The prevalence of internalized mental health problems are increasing from 15/16 to 18/19 years of age for both boys and girls, with an opposite trend for externalizing mental health problems. This is also in line with previous studies [32] and there might be a relationship between the reduction in externalized problems and prevalence of bullying [20].

3. For muscle and skeletal pain there is a diverging trend between boys and girls. While there is a prominent increase among girls for all pain types except abdominal pain, there is a simultaneous decrease among boys for all pain types with an increase in the number with no pain from 28.5 to 40.1%. There are only a few studies that have investigated somatic pain up to 19 years of age [21]. The conclusions from other studies, however, indicate that there is a fairly steep increase among girls and stagnation in the prevalence among boys [33].

4. Although a comparison of the different outcome measures is not justifiable due to different cut-off points, the strongest adjusted association between bullying, mental health problems and pain seem to be for internalized mental health problems at both15/16 and 18/19 years of age both for boys and girls. This finding has also been found in prospective studies. Cui and Vaillant followed up men from 26 to 65 years of age found that negative life events (including bullying) were statistically significantly associated with psychological, but not physical health [34] Also among adolescents similar results have been found [35]. The association also seems to go the other way around. In a six month follow up study in the Netherlands Fekkes et al found that 9 to 11 years old children that were depressed and had anxiety might be at an increased risk of being bullied [3].

5. The associations we measured were almost consistently stronger for boys compared to girls, although girls report more of mental health problems and muscle/skeletal pain. Other studies of negative life events have found stronger associations between internalized mental health problems and exposure to violence, especially sexual violation among boys than girls, but not for headache and neck/shoulder pain [36].

The impact of bullying can be looked at from different angles. One is to find possible increased utilization of health care services. The only statistically significant association found in the study by Haavet et al was that bullying is associated with more use of psychiatric/psychological services among boys, but not other primary health care services or hospital admissions [36]. This was in contrast to sexual violation which was associated with an increase in the utilization of all types of services among both boys and girls [36].

Another way to look at the impact of bullying is to study possible long-term effects on psychological health in adulthood. Fosse found in her doctorial thesis that there was a direct relationship between bullying at school age and serious mental health problems in adult life impairing the possibility to get higher education, being employed and engage in family life [37]. In Cui and Vaillant's follow up study of men experiencing negative life events in general they found an increased risk for developing affective spectrum disorders in later life [34].

### Strength and limitations

The strength of the study is the high response rate among the 15/16 year old. There was also a high response rate among the school enrolled participants among the 18/19 years old, but low for those enrolled by mail. This might be a source for selection bias. There are few missing data for the main questions applied in the young part of the HUBRO study. The measured Cronbach α for the HSCL-10 scale was high for all selected material. The Cronbach α for the SDQ, especially its subscales, was somewhat lower. The internal validity and reliability are therefore considered to be generally satisfactory.

The sample of 15/16 years old are probably representative for the adolescent population of Oslo and probably also for other multicultural cities in comparable countries. How representative the 18/19 old participants are, is more questionable. There are more non responders among immigrants and among boys. From a recent study Sagatun et al have shown that immigrants reported more mental health problems than ethnic Norwegians at age 15–16, and that the difference remained the same through the teenage years [38]. Adolescents are good informants on their health status and the mental health variables are well validated [28,39,40]. However, there is a possibility that bullying is understood differently in 15/16 and 18/19 year olds, and this might bias comparisons of the prevalence estimates in the two studies.

In addition to those already discussed there are four other major limitations of this study; the direction of causality, misclassification (including dependency in the data), lack of diagnostic validity and multiple outcome measures.

First, we have not been able to investigate the direction of events as we have only applied a cross-sectional design of the study. We had the possibility to analyse the data in a follow-up design, testing whether bullying at baseline has any effect on our outcome variables at follow up. Because of the strong dependency between bullying and our outcome variables and due to the fact that bullying is a persisting phenomenon both before and after the age of 15/16 years we chose to use the data as two cross sectional studies. A longitudinal analysis would also answer different questions. Our study design, therefore, does not allow for any causal interpretation.

Second, information bias is often present in cross-sectional studies especially non-differential misclassification. There might be responders (personality types) that systematically tend to report negative exposure and the most negative outcome or most positive exposure and outcome. A pupil with a depressive personality type might have a tendency to report more bullying, mental health problems and muscle/skeletal pain than a non-depressive personality type resulting in dependent misclassification. This might inflate (and in some cases deflate) associations falsely [41].

One way to solve this possible source of bias is to obtain objective information about exposure and/or outcome [42]. In our case, the information about the mental health problems and muscle and skeletal pain should have been obtained from parents or primary physician records. Recall bias might also have been a problem in this study. First, persons with mental distress may be more likely to report any type of trouble through a mechanism of selective recall of unpleasant events and bullying may certainly be such an event [43].

Third, the obvious lack of more detailed information about the bullying as well lack of psychiatric diagnoses and both intensity and duration of the muscle/skeletal pain severely hampers the validity of the findings. The measure we have used to study bullying is quite simple and do not include type, intensity and duration. This might affect the results both comparing prevalence figures and in the strength of the association with mental health problems. There might be great differences in the health impact of an adolescent teased by one person on the way to school compared to an adolescent that is suffering from systematic bullying with a prolonged duration. We can therefore only generalize this study to include adolescents reporting on the same simple measures of bullying.

Fourth, one of the problems incurred when calculating many main effects in a single study is the increased risk of identifying/generating "significant" results by chance. In this study we operate with seven outcome variables that we have tested at two time points stratified on gender. Conclusions based on significance testing should therefore be drawn with great care.

## Competing interests

The authors declare that they have no competing interests.

## Authors' contributions

LL drafted the manuscript and conceived the study. KG and AW-V performed the statistical analysis and helped to draft the manuscript. EB participated in the design of the study and coordination. All authors read and approved the final manuscript.

## Supplementary Material

Additional file 1 Table S2 – The cross-sectional associations between bullying and health complaints at 15/16 years of age. Table S3 – The cross-sectional associations between bullying and health complaints at 16/19 years of age. Table S1 – The table provides prevalence of mental health problems and different types of pain across gender and year of survey. Table S2 – The table shows the association between mental health problems, different pain types and bullying expressed as Odds Ratios. Table S3 – The table shows the association between mental health problems, different pain types and bullying expressed as Odds Ratios. Click here for file
