# Supplementary material for: Mental and somatic health complaints associated with school bullying between 10th and 12th grade students; results from cross sectional studies in Oslo, Norway
Source: Clin Pract Epidemiol Ment Health. 2009 Mar 23;5:6. doi: 10.1186/1745-0179-5-6 (PMC2667479; doi:10.1186/1745-0179-5-6)
Supplement: Additional file 1 — Table S2 – The cross-sectional associations between bullying and health complaints at 15/16 years of age. Table S3 – The cross-sectional associations between bullying and health complaints at 16/19 years of age. Table S1 – The table provides prevalence of mental health problems and different types of pain across gender and year of survey. Table S2 – The table shows the association between mental health problems, different pain types and bullying expressed as Odds Ratios. Table S3 – The table shows the association between mental health problems, different pain types and bullying expressed as Odds Ratios. [file 1745-0179-5-6-S1.doc]

**Additional file 1**

**Table S1 - Frequency of mental health problems and pain, number and percentage***

|  | Boys | | | | Girls | | | |
| --- | --- | --- | --- | --- | --- | --- | --- | --- |
|  | 2001 (N=1923) | | 2004 (N=1670) | | 2001 (N=1867) | | 2004 (N=2120) | |
|
|  | N | % | N | % | N | % | N | % |
|  |  |  |  |  |  |  |  |  |
| Internalized symptoms | 183 | 9,7 (8,3-11,0) | 230 | 14,0 (12,2-15,7) | 493 | 26,7 (24,7-28,7) | 716 | 34,5 (32,5-36,6) |
|  |  |  |  |  |  |  |  |  |
| Externalized symptoms | 266 | 14,1 (12,5-15,6) | 146 | 9,2 (7,8-10,7) | 193 | 10,4 (9,0-11,8) | 177 | 8,7 (7,5-10,0) |
|  |  |  |  |  |  |  |  |  |
| Headache | 846 | 45,8 (43,5-48,0) | 622 | 39,8 (37,4-42,2) | 1213 | 67,1 (64,9-69,2) | 1360 | 68,0 (66,0-70,0) |
|  |  |  |  |  |  |  |  |  |
| Pain in neck/shoulder | 511 | 28,0 (25,9-30,0) | 405 | 26,1 (23,9-28,3) | 752 | 42,8 (40,5-45,1) | 1041 | 53,1(50,9-55,3) |
|  |  |  |  |  |  |  |  |  |
| Pain in arm/leg/knee | 618 | 33,7 (31,5-35,8) | 349 | 22,4 (20,4-24,5) | 566 | 32,8 (30,6-35,1) | 648 | 33,6 (31,5-35,7) |
|  |  |  |  |  |  |  |  |  |
| Abdominal pain | 373 | 20,5 (18,6-22,3) | 223 | 14,4 (12,7-16,2) | 878 | 49,7 (47,4-52,1) | 928 | 47,4 (45,2-49,6) |
|  |  |  |  |  |  |  |  |  |
| Back pain | 601 | 32,3 (32,2-34,4) | 469 | 30,2 (27,9-32,5) | 670 | 38,3 (36,1-40,6) | 906 | 46,2 (44,0-48,4) |
|  |  |  |  |  |  |  |  |  |
| Number of pain sites |
| No pain | 548 | 28,5 (26,5-30,5) | 669 | 40,1 (37,7-42,4) | 273 | 14,6 (13,0-16,2) | 352 | 16,6 (15,0-18,2) |
| One or two | 944 | 49,1 (46,9-51,3) | 712 | 42,6 (40,3-45,0) | 859 | 46,0 (43,8-48,3) | 821 | 38,7 (36,7-40,8) |
| Three to five | 431 | 22,4 (20,6-24,3) | 289 | 17,3 (15,5-19,1) | 735 | 39,4 (37,2-41,6) | 947 | 44,7 (42,6-46,8) |

*Percentages are reported with 95% confidence intervals.

**Table S2 - The cross-sectional associations between bullying and health complaints at 15/16 years of age**

|  | | Boys | | | | | | Girls | | | | | |
| --- | --- | --- | --- | --- | --- | --- | --- | --- | --- | --- | --- | --- | --- |
|  |  | Crude | | | Adjusted* | | | Crude | | | Adjusted* | | |
|  |  | OR | CI L | CI U | OR | CI L | CI U | OR | CI L | CI U | OR | CI L | CI U |
| *Internalized symptoms* | Never bullied | 1,0 |  |  | 1,00 |  |  | 1,0 |  |  | 1,00 |  |  |
|  | Sometimes | 3,4 | 2,3 | 4,9 | 3,11 | 2,07 | 4,69 | 2,0 | 1,5 | 2,8 | 1,72 | 1,24 | 2,37 |
|  | Weekly | 8,4 | 4,0 | 17,7 | 4,34 | 1,86 | 10,13 | 4,7 | 1,7 | 13,3 | 4,79 | 1,62 | 14,19 |
|  | Several times a week | 13,3 | 6,9 | 25,7 | 8,68 | 4,12 | 18,29 | 3,1 | 1,6 | 6,3 | 2,08 | 0,97 | 4,46 |
| *Externalized symptoms* | Never bullied | 1,0 |  |  | 1,00 |  |  | 1,0 |  |  | 1,00 |  |  |
|  | Sometimes | 1,5 | 1,0 | 2,2 | 1,31 | 0,89 | 1,95 | 1,5 | 1,0 | 2,3 | 1,18 | 0,74 | 1,87 |
|  | Weekly | 4,0 | 1,9 | 8,2 | 2,15 | 0,95 | 4,88 | 1,3 | 0,3 | 5,9 | 1,28 | 0,27 | 6,02 |
|  | Several times a week | 3,7 | 1,9 | 7,3 | 2,31 | 1,07 | 5,00 | 2,4 | 1,0 | 5,7 | 1,26 | 0,50 | 3,18 |
| *Headache* | Never bullied | 1,0 |  |  | 1,00 |  |  | 1,0 |  |  | 1,00 |  |  |
|  | Sometimes | 1,5 | 1,2 | 2,0 | 1,47 | 1,09 | 1,97 | 1,9 | 1,3 | 2,7 | 1,69 | 1,18 | 2,41 |
|  | Weekly | 1,9 | 0,9 | 3,9 | 1,53 | 0,72 | 3,26 | 1,1 | 0,4 | 3,1 | 1,00 | 0,34 | 2,99 |
|  | Several times a week | 2,5 | 1,3 | 4,9 | 1,85 | 0,90 | 3,82 | 1,6 | 0,7 | 3,6 | 1,37 | 0,60 | 3,15 |
| *Pain neck/shoulder* | Never bullied | 1,0 |  |  | 1,00 |  |  | 1,0 |  |  | 1,00 |  |  |
|  | Sometimes | 2,1 | 1,6 | 2,8 | 1,83 | 1,35 | 2,50 | 1,5 | 1,1 | 2,0 | 1,47 | 1,07 | 2,02 |
|  | Weekly | 4,6 | 2,2 | 9,6 | 3,94 | 1,79 | 8,66 | 1,9 | 0,6 | 5,4 | 1,79 | 0,61 | 5,27 |
|  | Several times a week | 3,1 | 1,6 | 5,8 | 2,03 | 1,00 | 4,11 | 1,2 | 0,6 | 2,5 | 0,82 | 0,38 | 1,74 |
| *Pain in arm/leg/knee* | Never bullied | 1,0 |  |  | 1,00 |  |  | 1,0 |  |  | 1,00 |  |  |
|  | Sometimes | 1,6 | 1,2 | 2,1 | 1,46 | 1,08 | 1,97 | 1,8 | 1,3 | 2,4 | 1,71 | 1,25 | 2,35 |
|  | Weekly | 3,5 | 1,7 | 7,3 | 3,07 | 1,41 | 6,69 | 1,9 | 0,6 | 5,7 | 1,70 | 0,56 | 5,15 |
|  | Several times a week | 2,6 | 1,3 | 5,0 | 1,87 | 0,92 | 3,79 | 1,6 | 0,7 | 3,3 | 1,24 | 0,57 | 2,69 |
| *Abdominal pain* | Never bullied | 1,0 |  |  | 1,00 |  |  | 1,0 |  |  | 1,00 |  |  |
|  | Sometimes | 1,4 | 1,0 | 2,0 | 1,36 | 0,96 | 1,92 | 1,6 | 1,2 | 2,2 | 1,62 | 1,18 | 2,21 |
|  | Weekly | 2,4 | 1,1 | 5,0 | 2,24 | 1,02 | 4,90 | 2,0 | 0,7 | 5,9 | 2,06 | 0,68 | 6,23 |
|  | Several times a week | 3,3 | 1,7 | 6,3 | 2,41 | 1,18 | 4,93 | 3,5 | 1,5 | 8,2 | 3,02 | 1,26 | 7,26 |
| *Back pain* | Never bullied | 1,0 |  |  | 1,00 |  |  | 1,0 |  |  | 1,00 |  |  |
|  | Sometimes | 1,7 | 1,3 | 2,2 | 1,44 | 1,06 | 1,95 | 1,9 | 1,4 | 2,5 | 1,70 | 1,24 | 2,33 |
|  | Weekly | 2,2 | 1,1 | 4,6 | 1,56 | 0,72 | 3,38 | 0,7 | 0,2 | 2,2 | 0,61 | 0,19 | 1,99 |
|  | Several times a week | 6,1 | 3,0 | 12,4 | 3,85 | 1,83 | 8,14 | 1,3 | 0,6 | 2,6 | 0,92 | 0,43 | 1,96 |

* Adjusted for exposure to violence, having close friends, ethnicity, family structure, and parental socio-economic status (SES)

**Table S3. The cross-sectional associations between bullying and health complaints at 18/19 years of age**

|  | | Boys | | | | | | Girls | | | | | |
| --- | --- | --- | --- | --- | --- | --- | --- | --- | --- | --- | --- | --- | --- |
|  |  | Crude | | | Adjusted* | | | Crude | | | Adjusted* | | |
|  |  | OR | CI L | CI U | OR | CI L | CI U | OR | CI L | CI U | OR | CI L | CI U |
| *Internalized symptoms* |  |  |  |  |  |  |  |  |  |  |  |  |  |
|  | Bullied, but not last 12 months | 4,4 | 2,7 | 7,3 | 6,16 | 2,59 | 14,63 | 4,8 | 3,0 | 7,7 | 3,82 | 1,91 | 7,63 |
|  | Bullied incl last 12 months | 7,1 | 2,8 | 18,1 | 2,78 | 0,47 | 16,35 | 4,6 | 2,3 | 8,9 | 4,64 | 1,97 | 10,92 |
| *Externalized symptoms* | Not bullied | 1,0 |  |  | 1.00 |  |  | 1,0 |  |  | 1.00 |  |  |
|  | Yes, not last 12 months | 2,8 | 1,5 | 5,1 | 1,72 | 0,48 | 6,21 | 3,2 | 1,9 | 5,4 | 3,54 | 1,59 | 7,86 |
|  | Yes, incl. last 12 months | 5,5 | 2,0 | 14,9 | 7,24 | 1,08 | 48,41 | 1,6 | 0,6 | 4,1 | 1,01 | 0,31 | 3,30 |
| *Headache* | Not bullied | 1,0 |  |  | 1.00 |  |  | 1,0 |  |  | 1.00 |  |  |
|  | Yes, not last 12 months | 1,8 | 1,1 | 2,8 | 1,74 | 0,76 | 3,99 | 1,4 | 0,9 | 2,3 | 1,44 | 0,69 | 3,03 |
|  | Yes, incl. last 12 months | 5,6 | 1,8 | 16,9 | 8,31 | 0,96 | 72,04 | 1,7 | 0,8 | 3,6 | 1,42 | 0,59 | 3,43 |
| *Pain neck/shoulder* | Not bullied | 1,0 |  |  | 1.00 |  |  | 1,0 |  |  | 1.00 |  |  |
|  | Yes, not last 12 months | 1,9 | 1,2 | 3,1 | 1,90 | 0,82 | 4,39 | 2,1 | 1,3 | 3,4 | 1,62 | 0,82 | 3,23 |
|  | Yes, incl. last 12 months | 3,4 | 1,2 | 9,5 | 0,77 | 0,11 | 5,62 | 2,5 | 1,3 | 5,1 | 1,54 | 0,69 | 3,42 |
| *Pain in arm/leg/knee* | Not bullied | 1,0 |  |  | 1.00 |  |  | 1,0 |  |  | 1.00 |  |  |
|  | Yes, not last 12 months | 1,5 | 0,9 | 2,5 | 2,43 | 1,04 | 5,66 | 1,9 | 1,2 | 3,0 | 1,20 | 0,62 | 2,35 |
|  | Yes, incl. last 12 months | 9,5 | 3,4 | 26,9 | 2,36 | 0,48 | 11,69 | 2,5 | 1,4 | 4,8 | 2,59 | 1,20 | 5,60 |
| *Abdominal pain* | Not bullied | 1,0 |  |  | 1.00 |  |  | 1,0 |  |  | 1.00 |  |  |
|  | Yes, not last 12 months | 1,8 | 1,0 | 3,1 | 1,56 | 0,59 | 4,10 | 2,0 | 1,3 | 3,1 | 2,11 | 1,06 | 4,21 |
|  | Yes, incl. last 12 months | 2,3 | 0,7 | 7,2 | 0,30 | 0,01 | 5,96 | 2,0 | 1,1 | 3,8 | 1,52 | 0,71 | 3,27 |
| *Back pain* | Not bullied | 1,0 |  |  | 1.00 |  |  | 1,0 |  |  | 1.00 |  |  |
|  | Yes, not last 12 months | 1,8 | 1,1 | 2,9 | 2,00 | 0,86 | 4,64 | 1,9 | 1,2 | 3,0 | 1,76 | 0,89 | 3,47 |
|  | Yes, incl. last 12 months | 3,5 | 1,3 | 9,2 | 1,84 | 0,30 | 11,14 | 1,9 | 1,0 | 3,6 | 1,15 | 0,53 | 2,50 |

* Adjusted for exposure to violence, having close friends, ethnicity, family structure, and parental socio-economic status (SES)
